# A Review on Mental Stress Assessment Methods Using EEG Signals

**DOI:** 10.3390/s21155043

**Published:** 2021-07-26

**Authors:** Rateb Katmah, Fares Al-Shargie, Usman Tariq, Fabio Babiloni, Fadwa Al-Mughairbi, Hasan Al-Nashash

**Affiliations:** 1 Biomedical Engineering Graduate Program, American University of Sharjah, Sharjah 26666, United Arab Emirates; b00081299@aus.edu; 2 Department of Electrical Engineering, American University of Sharjah, Sharjah 26666, United Arab Emirates; utariq@aus.edu (U.T.); hnashash@aus.edu (H.A.-N.); 3 Department of Molecular Medicine, University of Sapienza Rome, 00185 Rome, Italy; fabio.babiloni@uniroma1.it; 4 College Computer Science and Technology, University Hangzhou Dianzi, Hangzhou 310018, China; 5 College of Medicines and Health Sciences, United Arab Emirates University, Al-Ain 15551, United Arab Emirates; f.almughairbi@uaeu.ac.ae

**Keywords:** mental stress, EEG, data analysis, connectivity network, machine Learning

## Abstract

Mental stress is one of the serious factors that lead to many health problems. Scientists and physicians have developed various tools to assess the level of mental stress in its early stages. Several neuroimaging tools have been proposed in the literature to assess mental stress in the workplace. Electroencephalogram (EEG) signal is one important candidate because it contains rich information about mental states and condition. In this paper, we review the existing EEG signal analysis methods on the assessment of mental stress. The review highlights the critical differences between the research findings and argues that variations of the data analysis methods contribute to several contradictory results. The variations in results could be due to various factors including lack of standardized protocol, the brain region of interest, stressor type, experiment duration, proper EEG processing, feature extraction mechanism, and type of classifier. Therefore, the significant part related to mental stress recognition is choosing the most appropriate features. In particular, a complex and diverse range of EEG features, including time-varying, functional, and dynamic brain connections, requires integration of various methods to understand their associations with mental stress. Accordingly, the review suggests fusing the cortical activations with the connectivity network measures and deep learning approaches to improve the accuracy of mental stress level assessment.

## 1. Introduction

Mental stress is one of the contributing factors to health problems. It is defined as the human body’s response, controlled by the sympathetic nervous system (SNS) and hypothalamus–pituitary–adrenocortical axis (HPA axis), to mental, physical and emotional stimuli [1]. This expression can be used with regard to internal (personality structure) or external (dealing with problems) matters triggering various physiological and negative emotional changes [2]. Literature defined three types of stress; acute stress, episodic stress, and chronic stress [3]. Acute stress is related to short-lasting exposure and is not harmful. Episodic stress happens when the stimulus is more frequent for a limited time [4]. Meanwhile, chronic stress is the most damaging, resulting from permanent and long-standing stressors [5]. Several studies have reported that mental stress has direct physiological effects leading to several diseases including stroke, cardiovascular disease, cognitive problems, speech distinctiveness and depression [6,7]. Moreover, stress affects the human body indirectly at different levels varying between skin conditions, eating habits, inadequate sleeping and decision-making [8,9,10]. Thus, researchers have developed various methods to assess the stress level in its early stages to avoid the negative consequences on health and performance. 

Assessment of mental stress is challenging because each individual experiences stress differently [11]. Besides, the reliability of evaluating mental stress depends on the method of assessment and analysis. Traditionally, stress is assessed using subjective methods. The most commonly used method is the self-report questionnaires [12] such as the perceived stress scale [13,14]. Many studies have established the questionnaire score and self-report rating or interview as ground truth to estimate the mental stress level. However, questionnaires are subjective and require the user’s full attention. As a result, individuals are not always aware of their genuine stress levels. Hence, the procedures, such as self-report questionnaires, may result in an inaccurate stress level measurement. Furthermore, they seem to be less informative than physiological measures. Researchers have identified several physiological measurements as stress indicators such as heart rate variability (HRV), electrodermal activity (EDA), electromyogram (EMG), blood pressure, pupil diameter, salivary cortisol and salivary alpha amylase [2]. Nevertheless, physiological markers can be influenced by many factors including mental stress. Cortisol level has been reported to be affected by circadian rhythm (i.e., its concentration changes throughout the day) [15,16]. In addition, a subject’s physical activity affects salivary alpha amylase level [17,18], and EDA is sensitive to skin disease and humidity [19]. 

Various neuroimaging techniques have been used to assess mental stress by directly or indirectly measuring the brain activity. These include functional near-infrared spectroscopy (fNIRS), electroencephalography (EEG) [20], positron emission tomography (PET) [21] and functional magnetic resonance imaging (fMRI) [22]. The EEG modality has some advantages such as high temporal resolution, low cost, and ease of use. Hence, it is the most used technique to analyze mental states including stress [23,24]. A typical EEG stress assessment method consists of two major parts: feature extraction and stress classification. There are three categories of EEG features: time-domain, frequency-domain, and synchronicity-domain features [25,26,27]. The time-domain features capture the temporal information using amplitude related to energy, variability, coefficient of variation, Hjorth feature, fractal dimension feature and higher-order crossing feature. On the other hand, the most used frequency-domain features are obtained from the EEG signal clinical frequency bands, delta (0.5–4 Hz), theta (4–8 Hz), alpha (8–13 Hz), beta (14–30 Hz) and gamma (30–50 Hz) [28]. These brain rhythms contain relevant information related to mental stress and other psychological disorders. The commonly used spectral EEG features include the power spectral density (PSD), differential asymmetry features, phase synchronization, phase lag index, directed transfer function and entropies [29,30,31,32]. In addition, the time-frequency features are obtained through the short-time Fourier transform (STFT), or discrete wavelet transform (DWT) [33,34,35]. The findings of subsequent studies on the usefulness of EEG signal analysis methods for the assessment of mental stress have been conflicting and impeding the development of further research. To resolve these difficulties, this work aims at conducting a comprehensive review of the state-of-art of the published EEG analysis methods on mental stress and to propose potential future research directions.

The rest of the paper is organized as follows. The materials and methods are described in Section 2, where the explanation of inclusion and exclusion strategy in addition to the variables of interest are reported. EEG pre-processing and the data analysis methods are presented in Section 3. Section 4 reviews the most common classifiers that have been used in quantifying stress levels. Section 5 shows the review results, including the relationship between EEG analysis methods and type of classifier and the variables can be considered in assessing mental stress. The discussion of the findings on the reviewed papers is described in Section 6. Finally, Section 7 and Section 8 summarize the main challenges and conclusion of the research in stress estimation-based EEG signal.

## 2. Materials and Methods

### 2.1. Search Strategy

**Figure 1:**
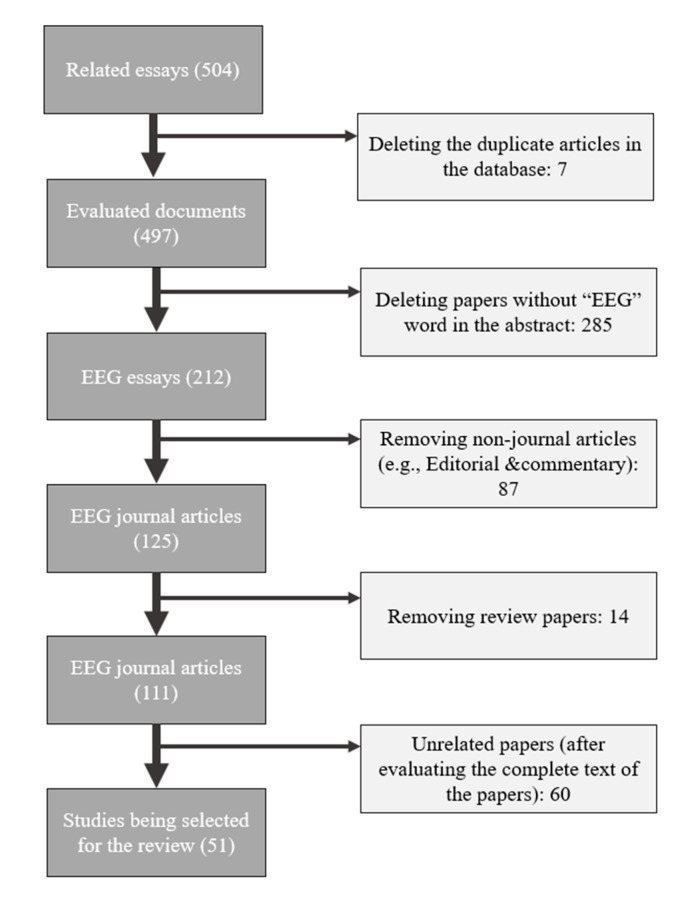
Flow chart of search strategy and identification of relevant studies.

The Preferred Reporting Items for Systemic Reviews and Analysis (PRISMA) was used to conduct this review [36]. The following databases were searched for study publications, namely Google Scholar, PubMed, Science Direct, IEEE Xplore, and PsycINFO. The used search terms were the single terms of mental stress and EEG. This was combined with at least one of the following terms: connectivity, power spectral, coherence, entropy and classification. In addition to searching databases, the reference list for all selected articles was checked to specify any additional relevant studies that might have been overlooked during the primary search. Figure 1 shows the search strategy and identification of relevant studies.

### 2.2. Inclusion and Exclusion Strategy

Manuscripts in English and EEG experimental studies were considered in this review. In contrast, those involving animals were excluded to avoid any possible effect of cognitive impairments.

### 2.3. Variables of Interest

The main variables detected in each paper were (i) type of stressor, (ii) experiment duration, (iii) number of subjects who participated in the experiment, (iv) number of EEG electrodes, (v) EEG frequency bands, (vi) type of features, (vii) type of classifier, (viii) classification performance, (ix) summary of results compared before and after the stress task, and (x) comments on the findings.

## 3. EEG Analysis Methods

The EEG signal goes through extensive preprocessing steps to remove artifacts and noise before applying data analysis methods. Data preprocessing plays a major role in getting meaningful information about the signal. Thus, comprehensive knowledge about the types of artifacts is required. According to Jiang et al. [37], physiological artifacts are the most common artifacts that affect EEG signal. In addition, artifacts represent another vital source of biased information. The digitized EEG signal can be segmented into epochs (e.g., 2 s) for visually identifying and rejecting visible artifacts. To remove the noise and artifacts from EEG signals, researchers have utilized a variety of methods such as regression techniques, blind source separation (BSS), empirical-mode decomposition (EMD) and wavelet transform algorithms. This is in addition to Adaptive and Wiener filtering, high pass, band pass, notch filters, and independent component analysis (ICA) [38]. In fact, we still have a lack of standardization related to EEG pre-processing that can be used by all research studies. 

In stress studies, the process of reviewing, cleansing, transforming, and modelling EEG signals with the aim of finding useful knowledge, informing conclusions, and assisting decision-making is known as data analysis. Several data analysis methods have been reported in the literature to analyze mental stress based on EEG signals. However, selecting an appropriate analysis method is very important to minimize the data processing cost, storage size and dimensional space. The following sub-section provides a comprehensive review of the EEG analysis methods on mental stress.

### 3.1. Connectivity Methods

The primary objective of EEG research is to link diverse measures of neural rhythms to functional brain states reflecting cognition, behavior, or neuropathology [39]. Each EEG signal is produced by the superposition of several brain current sources [40]. The involvement of each source varies depending on the location and orientation of the source and measuring electrodes. Several researchers have shown interest in functional or effective connectivity. However, the various forms of data used to assess functional connectivity differ in many ways, involving temporal and spatial information, as well as whether the data reflect electrical neuron activities, neuronal ensemble activities, or hemodynamics of macroscopic brain areas. Furthermore, the exact computational techniques employed to find these values vary amongst researchers, even when dealing with the same data type.

The issue is considerably more complex in the case of EEG, where numerous aspects of the signals might be linked. The information in an EEG signal comes from a complex and dense network of interconnected neurons. Hence, studying brain connectivity may provide us with a more exact model of the brain and how its various areas interact with each other. There are two types of brain connectivity: functional connectivity and effective connectivity [41]. The functional connectivity reflects the relationships between different brain regions as reflected on the temporal coherence between the networks. The various methods for determining functional connectivity may result in different conclusions depending on factors such as the strength of the interaction between neural units, type of stressor and number and location of electrodes. This can even be true for data from the same modality and even data obtained using the same task. Employing multiple interpretations of what defines functional connectivity might also lead to conflicting findings [41]. Effective connectivity, on the other hand, is the simplest circuit that describes the experimentally achieved relationship between two neurons. It explains how the neural system affects the others [42]. Effective connectivity, in contrast to the non-directional and correlative functional connectivity, assesses the directional influences between distinct brain regions [43]. As such, functional and effective connectivity measures are important in trying to understand the brain behavior under stress and non-stress conditions. There are numerous features utilized to detect this connectivity measurement, and the following is a quick description of them.

Coherence analysis aims to identify the functional connectivity and synchronization between different brain regions (several electrode sites). These mutual relations can be found by analyzing the amplitude and the phase of signal within the used EEG electrodes [44,45]. Xia et al. [44] examined coherence using multilevel stress assessment and found a significant increase for all frequency bands (except beta) at frontoparietal lobe. In addition, strong coherency for delta wave was detected in prefrontal and temporal regions at higher stress level. Meanwhile, study in [46] has shown an increased brain connectivity between interhemispheric locations in delta and theta bands, whereas the alpha and beta coherence connectivity networks spread all over the scalp. In particular, this increase in coherence level under stress in the article [46] was regarded as the brain attempting to attain redundant communication between its different regions in order to quickly process the cognitive load of the applied stressor. The full mathematical expressions of the coherence measures can be found in a previous study [47].

Magnitude Square Coherence (MSC) is another measure of functional connectivity in stress studies. A study in [48] found significant reduction in the functional connectivity from control to the stress situation in intra-hemispheric and inter-hemispheric prefrontal cortex (PFC). Meanwhile, when applying sleep deprivation as a stressor, the EEG connectivity maps show a decreased MSC for alpha band in the anterior region of scalp and increased beta coherence spread all over the scalp [49]. However, this behavior was not reproduced when dealing with Stroop color word task (SCWT) where there was only elevated beta coherence for sagittal middle regions. Darzi et al. [50] have proved that extracting MSC features with a length of 56 s achieved the highest accuracy by applying support vector machines (SVM) as a classifier compared to the directed transfer function (DTF), phase–slope Index (PSI), canonical correlation (CC), and power spectral density (PSD) techniques. Likewise, Khosrowabadi et al. [51], reported that MSC accuracy, sensitivity and specificity were greater than those obtained by Gaussian mixture models (GMM) and fractal dimension (FD) features using K-nearest neighbors (KNN) or SVM classifiers. Consequently, the most useful advantage about using coherence in analyzing EEG to quantify stress is that it cannot be affected by the amplitude oscillations for the different brain locations. However, the main drawback for coherence analysis is the high sensitivity to phase coupling and power changes [46,52]. The mathematical formulations of the employed MSC can be found in [53].

Pearson’s correlation-based captures linear, time-domain dependencies among EEG signals. It could be found over a single epoch or over several epochs, and it is calculated using the Pearson’s correlation coefficient, cross-covariance, and auto-covariance of EEG signals [54]. Therefore, increasing the value for the Pearson correlation coefficient from (−1) to (1) indicates intense connections between brain regions. In particular, this technique has been used by study [54] to reduce feature vectors and computational time, and to improve accuracy of SVM classifiers in detecting human stress. The main interest of such features is the high performance while reducing dimensionality of the EEG data set [55]. On the other hand, canonical correlation analysis (CCA) is useful to get information from the cross-covariance matrices in order to estimate the effect of mental stress. This is done by detecting the linear combination that achieves maximum correlation between two vectors [56]. The main advantage of using CCA is its applicability to be used with multimodal data that has different modal dimensionalities [57]. The mathematical expressions of the correlation analysis can be found in previous study [58]. 

Amplitude asymmetry refers to the difference in absolute amplitude that exists between the homologous electrodes positioned on the hemispheres when a stressor is applied. It is used to find the difference in the relative stimulation between brain locations [59]. Despite its high performance in estimating acute stress levels [44], this technique is influenced by HRV biofeedback [60]. The study in [61] describes the math of the asymmetry method.

Mutual information (MI) is used to detect dynamic concatenate and similarity of joint probability distribution function between two EEG signals [50,62]. Therefore, MI aims to find the statistical dependency between signals and analyze EEG with different spectral bands [63]. The MI during stress is represented by EEG connectivity maps. According to the study in [49], mutual information did not achieve significant increase in the EEG map when using Stroop task, whereas the sleep deprivation physical stressor showed widespread decreases of linear area comparing to a significant increase of nonlinear area in the anterior, central, and temporoparietal regions of head. Meanwhile, Pernice et al. [64] reported that the shared information between brain locations during relaxing state was low, whereas a significant MI increase was noted for alpha, theta and delta bands in the frontal region during mental arithmetic task. On the other hand, comparing to other connectivity measures, this technique has a short time processing and it is not restricted to real-valued variable; therefore, it could be used on several kinds of variables [65]. The mathematical formulations that describe MI can be found in [66].

Phase lag is used to detect the lag or delay between two EEG signals related to different brain regions. Xia et al. [44], has detected a significant role for the phase lag technique in discrimination between stress and control conditions for different levels. However, a study in [45] has found low accuracy for this feature compared to the other used methods such as coherence, absolute power and amplitude asymmetry. The main limitation of this technique is that it does not provide the directionality of connectivity and the volume conduction problem [67]. The mathematical expressions of the phase lag analysis can be found in previous study [68].

Phase–slope index (PSI) is a measure of phase synchronization that is not sensitive to volume conduction or common reference effects. Studies in [50] found several patterns of brain locations connectivity during the perception of external stimuli that chronic stress can change them, whereas the synchronization between left parietal and right temporal showed a decrease of 55% in the stressful subjects. Darzi et al. [50] have shown a high performance when using PSI features, whereas the results of Khosrowabadi et al. [69] achieved low accuracy for PSI comparing to DTF and PDC features. The main advantages of using PSI are overcoming the independent background activity generated between two electrodes and the ability to give meaningful information even though the nonlinear phase spectrum [69]. However, PSI may fail to correctly describe the directionality of EEG [70]. The mathematical formulations of the PSI method can be found in [71].

Partial directed coherence (PDC) is a measure used to detect the direction and weight of information flow in the frequency domain between multivariate data. Specifically, multivariate analysis will represent the stress phenomenon without loss of information of data with several variables. In particular, two directed coherences (feed-forward and feedback aspects) can be predicted from the classical coherence function using PDC. Therefore, directional flow between two channels within specific frequency involves several calculated factors such as Akaike information criterion and Granger causality [72]. Studies in [72,73] have found that when fatigue level increases due to stress, the functional coupling decreases over parietal-frontal regions while using theta, alpha and beta frequency bands. A significant form of PDC to get functional connectivity measurement is the Generalized Partial Directed Coherence (GPDC), which is used to control negative causality of the EEG multichannel analysis. Khosrowabadi et al. [69] has used GPDC features in detecting stress/non-stress cases, and they found medium and low accuracy compared to PSI and DTF features. The full mathematical expressions of the PDC measures can be found in a previous study [74].

Directed transfer function (DTF) is an effective connectivity technique used to detect the interaction patterns between neurons. Yu et al. [75] found that DTF has increased values (enhanced EEG coupling) at alpha and beta bands after applying a mental arithmetic stress task. In particular, these results lead to enhancing the flow of information from the central regions (the source of information outflow) to parietal and occipital areas for alpha and beta. According to a study [69] in quantifying stress, DTF shows the highest accuracy comparing to PSI and GPDC. However, DTF does not differentiate between directed influence of one signal to another [76], but it shows a higher performance than CCA, PSI, MSC and PSD [50]. Meanwhile, the main limitation for using DTF is its sensitivity to cortico-cortical and brain to heart functional coupling [75]. DTF mathematical expressions can be found in articles [77].

### 3.2. Power Spectral (Frequency Domain)

Spectral features are the characteristics obtained from the EEG signal in frequency domain. In order to get meaningful information about the EEG, it is important to check the segmentation process of EEG to get stationary signal. Thus, some of the more widely used spectral features and processing techniques are described below.

Power spectral density (PSD) pursues to find power distribution for time-domain EEG signal over frequency range and this provides significant information about cortical activation. In particular, PSD is useful in describing stochastic process of the signal and evaluating short data records [78]. There are several methods applied to estimating the PSD, for example, fast Fourier transform (FFT), Welch, Burg, Yule walker, welch method and periodogram [54]. Several studies have demonstrated the effectiveness of using PSD to estimate the level of stress. For example, study in [79], reported that mental stress decreased the EEG power spectral density in the alpha band. Likewise, the study in [20] found a significant decrease in alpha rhythm when increasing the level of stress from level 1 to level 2 (based on increasing the complexity/difficulty of the math task), and then increasing from levels 2 to level 3. In particular, the difficulty of the math task was increased from level 1 up to 3 by increasing the integer numbers and operands that were used in the math operation. Meanwhile, according to [20], the most dominant cortical structure that is involved in stress detection is the right prefrontal cortex. For detailed mathematical formulations of the PSD method, refer to [80].

Other studies utilized absolute power (AP) as an indicator of stress. The AP at a particular band is calculated by dividing the absolute value of fast Fourier transform of the EEG signal by the signal’s length [81]. Meanwhile, studies in [59,82] used the relative power (RP) to check the rhythm of EEG signal by finding the ratio between the power of each band and the power of the total bands. Subhani et al. [45] and Arsalan et al. [83] found that applying AP on stress/non-stress detection shows a significant difference regarding theta EEG band (4–7 Hz) compared to other bands, whereas in the case of RP, they reported that when stress levels increased, the RP decreased [45]. Consequently, RP showed a better performance compared to the AP in spite of its sensitivity to the noise and memory recall [81]. The detailed math expressions for the AP and RP methods are identified by study [45].

Studies in [26,79] utilized powers from the wavelet transform (WT) coefficients to extract features that are highly correlated with mental stress. They found that the mean alpha rhythm power has significantly decreased from one stress level to the next higher one. Moreover, WT is an appropriate method for multi-resolution time-frequency analysis. This is done by decomposing the EEG signal into its frequency bands retaining information in both: frequency and time domain. Then, from wavelet coefficients, the average power and energy can be estimated. Even though the Fourier transform (FT) provides a frequency domain representation of the signal, the wavelet transform creates a time and frequency domain representation, providing a quick access to the localized information of the signal. In particular, since EEG signals are nonstationary, using the FT may result in tiny changes in the spectrum, and the analysis may alter depending on the duration of data. Thus, WT is preferable to FT [84]. The mathematical formulations of the employed WT can be found in [85].

Other studies used Gaussian mixtures of EEG spectrogram to detect stress by analyzing the changing of spectral density of the EEG signal related to time domain. Moreover, this data analysis method involves short-time Fourier transform (STFT) to calculate the spectrogram of the time signal. After computing spectrogram, Gaussian mixture model (GMM), which is a linear combination of Gaussian pdfs, can be estimated to find the density [51]. The obvious role of this model is extracting the symmetric and asymmetric EEG signal; however, some drawbacks of considering infinite range and symmetric nature are reported [86]. Khosrowabadi et al. [51,87] have used this technique to quantify chronic mental stress. They found that GMM has a lower accuracy than MSC, but higher than FD features when using SVM classifier. The detailed math expressions for the Gaussian method are identified by studies [87].

The study in [88] quantified mental stress by using spectral moments (SM). SM was processed to detect three power spectral moments from each EEG segment, that are related to different root square moments with orders of zero, two and four. These moments are found depending on the phase excluded power spectrum and the EEG length. Attallah in [88] verified the effectiveness of spectral moment in differentiating stress/non-stress cases and between several stress levels and reported high accuracy for SM with a linear discriminant analysis (LDA) classifier. The full mathematical expressions of the SM method can be found in [89].

### 3.3. Time Domain Techniques

The most widely used temporal features in quantifying mental stress are reviewed below:

Hjorth parameters are statistical parameters used to describe the EEG signal in the time domain. The Hjorth parameters are also known as normalized slope descriptors (NSDs). They consist of activity, mobility, and complexity descriptors. Activity parameter demonstrates the signal power leading to denoting the surface of the power spectrum in the frequency domain. The mobility approximates the mean frequency, and complexity approximates the bandwidth of the signal [90,91]. These parameters depend on the time domain, but they provide information about the frequency spectrum of the EEG [92]. However, theses parameters are sensitive to noise. Besides, the Hjorth parameters need shorter computation time in getting frequency information in addition to forming a good alternative for short time Fourier transform (STFT). Oh et al. [93] found that combining Hjorth parameter with band pass filtering has a higher classification performance than the general Hjorth parameter. The mathematical formulation of the employed Hjorth parameter can be found in [94].

Other methods to estimate the complexity of EEG signals in the time domain are the entropies. For example, Shannon entropy (SE) is used to estimate EEG signal irregularity and to quantify energy distribution of power spectrum by analyzing the EEG time series. This leads us to know brain behavior during a variety of states to detect mental stress [95]. Therefore, the study in [95] found the group that had the highest stress index (high mental stress) tend to have the lowest alpha-band-entropy. Zhu et al. [96] used VR-based relaxation therapy to relieve stress by evaluating the changes in Shannon entropy. They reported that SE had an increased trend in the alpha band, before and after watching VR. Another type of entropy is the Approximate Entropy (ApEn), which is used with time series data to know the fluctuations unpredictability and the amount of the regularity. According to Wang et al. [97], the complexity of the system is responsible for determining data length when estimating the value of ApEn. Meanwhile, the study in [97] showed that mental arithmetic task induced a significant increase of ApEn at the anterior cingulate and insular cortex. The main advantage for ApEn is its ability to deal with noise and possibility to be used with stochastic and deterministic chaotic signals. Moreover, the wavelet sum of entropy was utilized by Hasan et al. [90] as a separate feature to identify the signs of stress from EEG recordings. It represents the summation of the entropy after being calculated for each wavelet band. These wavelet bands can be found as a result of dividing EEG signal onto distinct frequency bands (generally five bands) and applying discrete wavelet transform (DWPT) [90]. Finally, self-entropy (SE) is used to detect information processing within the physiological network by estimating dynamical activity of the EEG signal [19,62]. Studies [96] include the mathematical expression for all entropy kinds.

Higuchi’s fractal dimension (FD) is the estimation of irregularity, complexity, and nonlinear properties of the EEG signal where high and low values of FD are related to irregular and regular waveforms, respectively [11]. Higuchi FD provides a significant analysis for stress phases by computing fractal dimension, which is useful in real-time testing for brain chaotic behavior during chronic mental stress [51]. Studies in [11,98] have shown that combining FD with statistical features outperforms spectral power features. The recorded EEG complexity in frontal lobe has high values when using mental arithmetic stressor [98]. On the other hand, Khosrowabadi et al. [51] detected a low accuracy for Higuchi’s FD comparing to GMM and MSC features for SVM and KNN classifiers. The main interest about FD is its independency of signal nature and high efficiency, but it is sensitive to noise and frequency bands and its performance will be low when it is used alone [99]. The mathematical formulations of the employed FD can be found in [100].

### 3.4. Statistical Features

This type of features can be found by applying standard statistical operations on the EEG signal within the time domain to quantify stress levels. Thus, statistical techniques are simple, easy to use and often complement each other [101]. Meanwhile, the most common features for EEG data analysis are the mean, skewness, kurtosis, standard deviation, shape factor, first and second difference, root mean square, and impulse factor [88,90,92,102]. Hou et al. [11] found that combining statistical features with fractal dimension and power features improved the classification accuracy of stress. Moreover, study in [103] found that the variance values are higher in rest than stress levels, whereas kurtosis showed increased values in stress conditions when moving from delta to gamma bands. On the other hand, the main drawback is related to using all these features in stress estimation, which leads to longer time processing. Furthermore, some studies utilized principal component analysis (PCA) as a conventional and statistical method for detecting samples in the EEG data of high dimension. According to Deshmukh et al. [104], the main purpose of using PCA was to reduce the dimension of the stress features before feeding into the classifier. This is done by applying features Eigen vectors on features dimensionality to get the lowest orthogonal dimensions [44]. Moreover, PCA provides information about how the investigated groups, related to stress/non-stress conditions, could be separated into principal components (PCs) space [105]. Shon et al. [92] analyzed mental stress and demonstrated that PCA has a lower accuracy (65.30%) in the process of features selection than genetic algorithm (71.76%). However, PCA limitation is the probability to fail in processing data when dealing with complicated manifold [104].

## 4. Classification

Stress studies have examined various types of classifiers to assess the level of mental stress. The most common and significant classifiers are SVM, LR, NB, KNN, LDA, multi-layer perceptron (MLP), convolutional neural network (CNN) and long short-term memory (LSTM). The following sections describe the implementation of the aforementioned classifiers on EEG stress studies. Table 1 summarizes the main findings of previous EEG stress studies. 

SVM is a binary classification model built in feature vector to discover the hyperplane that optimizes the margin between input data classifications. Several studies used SVM to discriminate between stress levels. For example, the studies in [51,106] applied SVM to quantify two levels of stress and achieved accuracy levels of 75% and 90%, respectively. On the other hand, studies in [107] have utilized SVM to classify three levels of stress. Meanwhile, [26] combined SVM with an error-correcting output code and reported that the average classification accuracy of these mental stress levels showed a drop in value from 97.61 to 95.37 and to 91.40 with the increased stress level. Besides, Gaikwad et al. [107] had an accuracy of 72.30% in the real time by using a trained algorithm as a reference. According to Hou et al. [11], increasing the number of stress levels (from two levels up to four) declined the SVM accuracy. 

Furthermore, studies in [19] have utilized LR to differentiate between stress levels. LR is a statistical model that utilizes a logistic function to represent a binary dependent variable in its most basic form, however many more advanced extensions exist. It is used to investigate the relationship between one dichotomous dependent variable and one (categorical or continuous) independent variable. Zanetti et al. [19] analyzed three mental states and the recorded accuracy by LR was 84.30%, but even though LR had some errors in detecting resting states. Meanwhile, the achieved accuracy by LR was as high as SVM and random forest classifiers when it was used with several stress states induced by arithmetic stress task [45]. Saeed et al. [108] showed that logistic regression provides a significant performance with 85.15% accuracy in stress quantification (specifically with alpha asymmetry feature) comparing to other classification techniques such as KNN, NB and MLP.

Some studies employed NB to classify stress levels. NB is a simple and fast probabilistic classifier that is used when input dimensionality is large. It is based on Bayes’ theorem, which assumes that extracted features are independent to each other. Subhani et al. [45] reported that NB achieved the highest accuracy in quantifying four levels of stress with a recorded accuracy of 94.0%, 94.6%, and 91.7% for levels 1, 2, and 3, respectively. Darzi et al. [50] detected two levels of stress using NB and found that SVM has a better performance than NB even though the running time of NB is about five times shorter than SVM, therefore NB is more suitable for online tasks. Thus, NB provides fast stress quantification because no complex optimization parameters are required. Meanwhile, NB had a low accuracy in Arsalan et al. [83] when dealing with theta band of two stress levels (75%) and three stress levels (50%). Moreover, Saeed et al. [108] recorded an accuracy of 80.79% for quantifying stress by NB, whereas in [109] they showed that using low beta waves as a feature vector will reduce NB performance to get an accuracy equal to 71.4%.

Furthermore, the non-parametric learning algorithm K-NN can be involved in quantifying mental stress. The mechanism of K-NN depends on estimating the distance between neighbors and choosing the K closest neighbors. Thus, two of the critical factors to be identified are the optimal value of K and neighbors distance D [90,108]. Saeed et al. [108] used K-NN with alpha asymmetry, beta, and gamma waves as features to quantify long-term stress. They found that K-NN has an accuracy of (65.96%) when these features are combined with each other. Meanwhile, the study in [50] found that K-NN has achieved an accuracy of (90.0%) comparing to the SVM and Bayesian classifiers. The main advantage of K-NN is the low computational complexity in quantifying stress/non-stress phases when dealing with small-sized data [50,90]. However, K-NN has a drawback, which is the high sensitivity to data local structure (dimensions). 

On the other hand, some studies applied LDA as a machine learning method to classify stress by finding the linear combination between EEG features. Therefore, it is difficult to apply LDA on nonlinear EEG data due to LDA’s linear nature [110]. LDA was applied by Minguillon et al. [111] to quantify three levels of stress using the average relative gamma as a feature and found that, increasing the number of stress markers will enhance the value of the recorded accuracy (50.0%). Meanwhile, Vanitha et al. [112] found that LDA has the lowest accuracy (70.166%) comparing to the SVM (89.07%) and K-NN (72.67%) classifiers when detecting stress levels for students. Consequently, the main drawback of LDA is the assumptions and restrictions (linear decision boundaries) that are needed to establish this classifier [111]. 

Besides, MLP is a non-linear artificial neural network model that is used to map the input data into output data. It consists of multiple layers (at least three) that vary between input, output and one or more hidden layers. Since MLPs are fully connected, each layer is connected to the next one and each node will be as a neuron that uses non-linear activation function. Several studies have employed MLP to quantify mental stress. Saeed et al. [108] reported that, integrating alpha, beta and gamma features with MLP provides the highest accuracy (85.13%) compared to the one that can be achieved using a single feature. Meanwhile, Arsalan et al. [83] found that MLP outperforms both SVM and NB classifiers and gives the highest accuracy for both two-and three-class quantification of mental stress. Even though, the main drawback of MLP is the formation of over-fitting because of excessive or insufficient neurons [108].

Another example for deep networks is that of deep CNN, which is considered as a regulated MLP. It provides an alternative form to mimic the brain functionality in quantifying mental stress [23]. Comparing to the other classification algorithms, CNN needs a little pre-processing, can be used for large size nonlinear data and it provides a significant feature discrimination [113]. The main advantage of using CNN is the independence from human effort and prior knowledge. Several studies utilized CNN to analyze mental stress. For example, Jebelli et al. [114] quantified three levels of workers’ stress where CNN yielded an accuracy of 79.26% that outperforms SVM’s accuracy (79.12%), whereas in the study [115], CNN’s accuracy was equal to 86.62%. Meanwhile, they found that the optimum network configuration to quantify workers’ stress level needs two hidden layers with 83 and 23 neurons in the first and second hidden layers, respectively. Therefore, CNN facilitates the need for EEG feature extraction, which consumes time in the supervised learning algorithms [115].

## 5. Results

**Figure 2:**
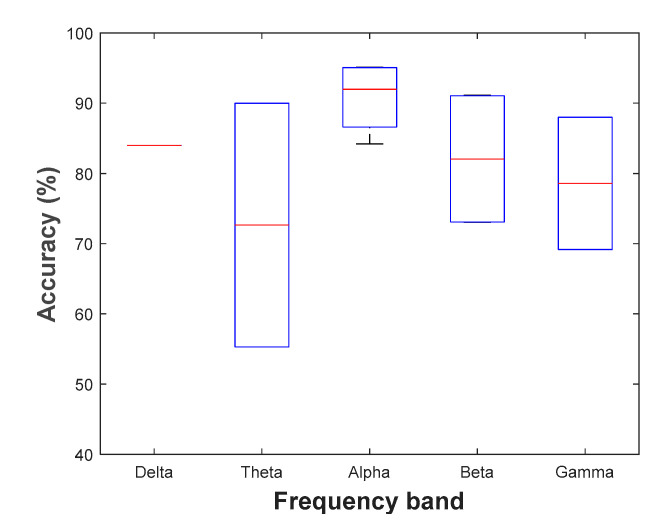
Classification accuracy based on EEG frequency bands.

Most of the reviewed studies have reported high alpha activity during relaxation states compared to the stressful conditions. In particular, a significant increase in the spectral power is more apparent after applying stimulus. EEG gamma activity showed a varied response, but generally a relatively decreased gamma activity can be observed with both relaxed and stressful situations. Hence, gamma oscillations may not be sensitive to stress level variations. Regarding fast beta band, it has a significant positive interaction indicating stronger increase in stress phases. Furthermore, central, and parieto-temporal areas are the most affected cortical regions with alpha and slow beta. Inspection of these variations related to different frequency bands were sided by the result of having stronger interaction effects in the right hemisphere comparing to the left one. Figure 2 summarizes the classification accuracy for each of the five different frequency bands extracted from the reviewed studies.

In general, accuracy refers to the percentage of accurate predictions. A value close to 100 indicates that the classification model is performing well. As a result, features are chosen from those EEG frequency bands that improve classification accuracy. To choose the best frequency band, all possible combinations (from five frequency bands) were used. According to the discussed sections, different classifiers were used to quantify mental stress using EEG. In order to get the proper performance, there are three parameters that will be needed: accuracy, sensitivity and specificity. They have been used to identify the classifier ability in correctly distinguish between positive and negative results and to measure each one of them properly. This performance is influenced by the quality of EEG signal, processing power and the EEG feature components that are used as an input to the classifier [83,116]. Arsalan et al. [83] found that, combining MLP classifier with PSD, correlation and rational asymmetry features outperforms SVM and NB in classifying two/three levels of stress. Furthermore, combining several results for multiple sensors may provide a better classification accuracy [117]. In particular, specific features and classifiers have reached high levels of accuracy such as PSD and SVM. Meanwhile, all references that have used Montreal Imaging Stress Task (MIST) as a stressor, rely on SVM classifier except Minguillon et al. [111], which has LDA instead. Despite the achieved low accuracy (50.00%) by the article [111], EEG measurements provided shorter response time, significant cognitive information and low sensitivity to physical activity. Thus, combining EEG with physiological signals elevates the LDA accuracy up to 86.00%. On the other hand, Xia et al. [44] got an accuracy equal to 79.45% when using ECG and EEG measurements with SVM classifier in addition to high number of participants and EEG electrodes. Therefore, the selected EEG features (relative power, power ratios, amplitude asymmetry, coherence, and phase lag) have shown promising and robust results when employed with MIST stressor and SVM classifier in quantifying mental stress. Furthermore, using NB classifier and the increased number of frequency bands were the main reason of getting high accuracy by Subhani et al. [45] comparing to Xia et al. [44] that have used the same criteria.

**Figure 3:**
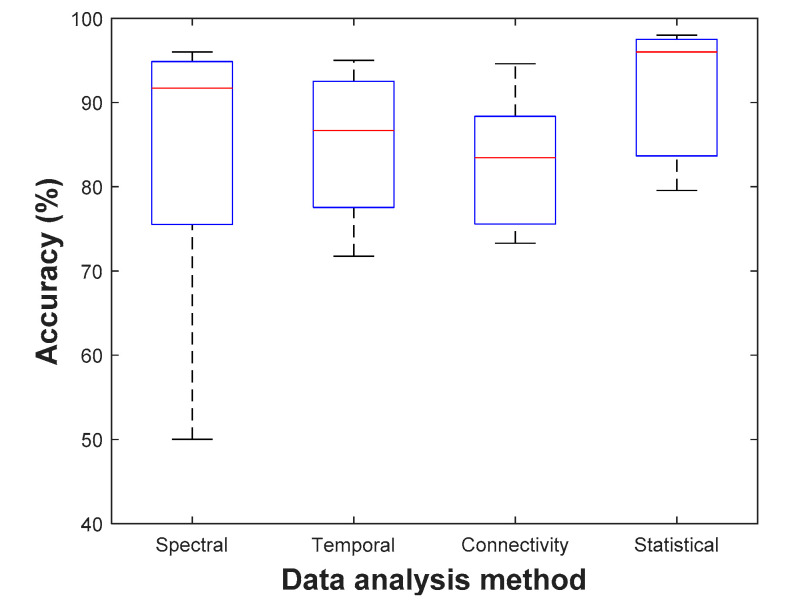
Classification accuracy with MIST stressor.

**Figure 4:**
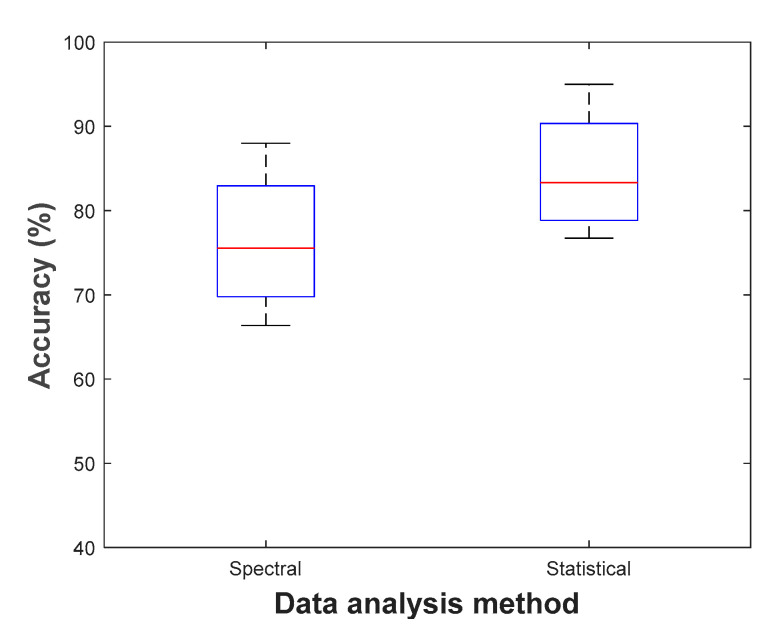
Classification accuracy with SCWT stressor.

Different stressors can be employed to generate mental stress, resulting in a variety of impacted brain regions. Students’ examination periods can be used to develop long-term psychological mental stressors. According to Darzi et al. [50] long-term stress affects the functional connectivity of the temporal-parietal and the left central and temporal regions. Furthermore, for music and videos stressors, pre-frontal region of the brain has shown increased activities when using two EEG electrodes to get differences between two frontal regions [92]. Lotfan et al. [118] utilized the Trier Social Stress Test (TSST), which includes free speech and mental arithmetic task in front of an audience, to induce moderate psychosocial stress. The brain connectivity measures revealed that the two situations, including before and 20 min after the TSST exposure, produced the same levels of stress. This indicates that the persistence of stress after 20 min fades and the brain network mimics the condition before stress. Al-Shargie et al. [119] used MIST, which increased beta rhythm power and decreased alpha rhythm power in the right pre-frontal cortex (sensitive to mental stress) and this is what was estimated by fMRI studies [120,121]. Likewise, using MIST task, the ventrolateral prefrontal area (VLPFC) achieved a higher accuracy than other PFC subregions [56]. Stroop color word task affects the temporal and spatiotemporal regions where several stress levels are induced individually to each subject [11]. For Maastricht Acute Stress Test (MAST), its protocol induces a realistic stress reaction in the subjects, which leads to variation of several salient physiological features [105]. Finally, driving task shows increased cortical activities for low level of stress, but it decreases with elevated stress level and time. Hence, this test makes a drop in alpha rhythm power when moving from rest to the stress state [122]. Figure 3 and Figure 4 compare the resulted classification accuracy of different types of EEG data analysis methods using MIST and SCWT stressors, respectively.

Some experiments of stress detection combined more than one stressor such as arithmetic task with either Stroop test [102,123] or relaxing videos [19] and mental workload with public speaking [124]. Moreover, as discussed by studies in [19,124], employing normal four frequency bands showed accuracy levels of 83.33% and 84.30% using NB and RF classification methods, respectively. However, Ahn et al. [123] derived two frequency fields (low and high bands) and reached 77.90% accuracy by SVM whereas the accuracy of Jun et al. [102] was about 96% by three different bands (theta, alpha, and beta) with SVM classifier. 

For studies that are interested in analyzing stress in normal daily life (psychological labelling), no stressors were introduced to the subjects. They used the same procedure in labelling participants and acquiring EEG data. There was an obvious variation in the treated frequency ranges. Thus, the highest accuracy was acquired when dealing with seven bands where they got 85.20% for SVM [108] comparing with the three bands 78.57% [54] and four bands 83.33% [106] that have used same classifier. Besides, the lowest performance was related to two frequency fields with 71.4% accuracy with NB classifier.

There are significant accuracies that have been achieved related to variety of stressor types. Studies in [118,124,125] used four bands but different classifiers and stimuli; for example, Lotfan et al. [118] obtained an accuracy of 92.31% with SVM and TSST stressor and noted increasing levels for another physiological measurement, which was cortisol level, whereas Masood et al. [125] detected 87.50% performance when applying CNN classifier and cognitive tasks, but Secerbegovic et al. [124] got a low value of 77.08% for SVM and mental workload test despite detecting a critical positive effect for applying EDA and ECG with the used EEG. 

Another set of studies examined the temporal lobe when having stressors as a form of odor and traffic noise. They found a positive correlation between mental stress and EEG beta power rhythms [126,127,128]. Table 1 summarizes previous studies related to mental stress classification using EEG signal. The summary focuses on the type of techniques that are used to quantify mental stress taking into consideration the number of subjects, number of EEG channels, type of stressor, duration of the experiment, the analyzed frequency band, the extracted features, type of classifier, and the achieved performance. The summary in Table 1 orders the reviewed studies based on the type of stressor. 

## 6. Discussion

Stress has become a growing problem in our daily lives by having a negative impact on both individuals and society. Different systems of the human body, such as the nervous, immune, cardiovascular, and gastrointestinal systems, are negatively affected by stress. This directly influences or transforms the hippocampus, a brain field, regardless of the nature of the stress. The victim’s memory and decision-making capabilities are harmed as a result of this brain alteration. It also has a detrimental effect on hormone excretion, which is important for proper immune system processing. Stress also causes cardiac-arrhythmias by amplifying or decreasing heartbeats, blood pressure, and creating disturbances in the cardio-vascular system. Meanwhile, it has negative effects on the gastrointestinal (GI) system, such as decreased appetite, disruption of normal GI tract activity, and crabby-bowel-syndrome. Thus, mental stress evaluation and analysis are very important procedures that can be done to detect stress in order to prevent significant health problems. Despite the number of studies that covered this phenomenon using EEG signals, there is a lack of inclusive guidelines about the relevance between EEG feature and its extraction methods. Here, we conducted a comprehensive review on the methods of analysis of mental stress-based EEG signals. Specifically, our review focused on the type of the method used for data analysis and classification model. In particular, we found that selecting the right method of analysis is challenging because of factors variety that are exercised in the experiments. These factors include EEG sensor, sample size, stressor type, task duration, time of the day, proper EEG processing, feature extraction mechanism, number of features and type of classifier. Therefore, the significant part related to mental stress quantification is choosing the most appropriate features. Another case of concern is the large discrepancy between individuals and response to stress. For example, different stress response may be acquired for a particular subject depending on his psychology, sociality, health, and emotional state. 

The methods of quantifying mental stress using EEG varies across the analysis spectrum. As previously stated, because the brain acts in networks, descriptors of network functioning will be required to completely comprehend neural processing. In this work, we provided a comprehensive review on these analysis methods. Meanwhile, we highlighted the key differences spotted between the research findings and argued that variations of the data analysis techniques could be a significant contributing factor towards several contradictory results. Besides, the extracting features that are related to brain connectivity showed a clear model of the brain and how its different regions are interacting with each other. Therefore, studying feature extraction techniques related to brain connectivity provides a clear model of the brain and how its different regions are interacting with each other.

Moreover, there is a variety in the experiment duration between the discussed references. Thirty minutes process of the study in [111] involves maximum voluntary contraction (MVC), resting state (RS), MIST training and task, three questions about self-perceived level of stress and a relaxation period. Meanwhile, the eighty minutes period of data acquisition of the studies [44,45] comes from two conditions (stress and control) where each one consists of 40 min of habituation, rest, four levels main condition, and recovery periods. In the experiment protocol of Al-Shargie et al. [20], it takes 60 min duration divided between introduction, training, resting, and the main experiment, which needs about 40 min using three levels of mental arithmetic task. Besides, the four minutes of the study in [88] depend on the experiment procedure, which includes 3 min of counting and 1 min of serial subtraction where EEG data is recorded. They have used 18 min duration in the study that involves a brief introduction, training, data recording for control and stress conditions. Saeed et al. [108] achieved an increase in beta rhythm power and a decrease in alpha rhythm power in the pre-frontal cortex with a total duration of 25 min. Consequently, to avoid the effect of time on subject’s cognitive ability and the influences of circadian rhythm on stress performance, it is preferred to conduct the EEG experiment on all participants at the same time of day [44].

The task nature and sample size had a direct influence on classifier accuracy, such as the restricted duration in doing mental arithmetic tasks, which leads to low performance accuracy [45] and the varied results gained with a large number of participants with the studies in [44,45] compared to [111]. It is worth noting that the majority of studies have a limited sample size, meaning that the amount of people involved is insufficient to overcome prejudices caused by individual differences. A larger sample size is needed to ensure statistical power and to bolster our findings. 

On the other hand, decreasing the number of EEG electrodes maintains real time stress detection, but could increase system mobility and ease. Therefore, using one or two frontal electrodes might be sufficient to detect stress/control phases, but in order to get their level it is better to use more electrodes as suggested [88]. As mentioned in the EEG processing section, the extracted EEG signal undergoes to several denoising processes that may eliminate the unwanted peaks and artefacts, but small remaining noise could deform the information of analyzed EEG.

Finally, the used EEG sensor to record data and measure mental stress has a huge impact on the number of channels available. The number of channels in a typical EEG system can range from 1 to 256. The 10/20 system, which governs the positioning of electrodes on the brain, is followed. The benefits of multi-channel EEG systems are that they do a better job of avoiding data loss, particularly as the sensor network expands to more channels (caused by when electrode distances grow further apart when fewer are deployed) as well as in detecting vital clinical signals. This means that medical applications need higher resolution EEG systems (larger sensor networks) to complete the task.

## 7. Challenges and Future Work

Most of studies induced stress in controlled environments, whereas the better method is to develop a protocol that sustains the real scenarios such as virtual reality. Furthermore, the discussed researches did not correlate the physiological changes, such as cortisol levels, with the behavioral response. Most of the reviewed studies conducted offline experiments, but we suggest developing an online system that deals with stress recognition in the real time. Moreover, one of the critical factors that influences stress assessment results is the ground truth that is needed to train the classifier by sorting subjects into stress/non-stress groups. Most of studies established this labelling by questionnaire score, psychologist interview or both of them. Nevertheless, these two methods cannot provide a direct judgment on mental stress existence because of the high dependency on participants themselves (in many cases, they expect a wrong stress situation because of subconsciousness). Unlike the used simulated experiments, a significant challenge will be faced when labelling subjects in real world tasks.

As a future work, suggesting EEG feature extraction techniques could be useful in improving stress detection such as phase synchronization and source localization. Phase synchronization is used to analyze interdependence between two-time EEG signals regardless of their amplitude. It has high sensitivity that leads to detect dynamical changes of brain functions during mental stress. While EEG is a powerful tool for measuring neuronal activity and connectivity, the lack of spatial resolution could be a drawback. EEG source localization may be used to estimate the locations of electrical activities from the scalp potential measurements. The information of localization about these active sources (depending on the recorded potential from the electrodes) provides a good diagnosis for the mental state and brain abnormalities. This method can be combined with other feature extraction techniques such as directed connectivity measures.

## 8. Conclusions

In this paper, we have presented a comprehensive review of EEG signal analysis methods for the assessment of mental stress. A rigorous procedure was adopted for the search strategy and identification of relevant studies. The review emphasized the major discrepancies between the research findings. It also suggests that various data processing methodologies have contributed to numerous conflicting outcomes. These various can be attributed to a number of variables, including the lack of a consistent procedure, brain regions of interest, type of stressor, duration of experiment, EEG signal processing, feature extraction technique, and the type of classifiers used. In addition, we have reported the effect of sample size bias in connectivity estimation. This problem can be solved by equalizing sample sizes between different conditions or participants, using statistical methods that explicitly account for sample size bias, or employing connectivity approaches that are not affected by sample size bias. Moreover, understanding the relationships between mental stress and the complex and diverse EEG characteristics, such as time-varying, functional, and dynamic brain connections, necessitates the integration of several data analysis methods. As a result, we propose combining the network connectivity measures with deep learning to increase the accuracy of assessing mental stress levels.

## Figures and Tables

**Table 1:** Previous studies related to mental stress classification using EEG signal.

Reference Number	Year	Number of Subjects	Number of Channels	Type of Stressor	Condition Types	Brain Regions	Experiment Duration	Frequency Bands	Features	Classifier	Performance Acc: Accuracy Spec: Specificity Sen: Sensitivity	Notes
[129]	2020	25	4	MIST	Relax–Stress	PFC	24 min	4–13 Hz 8–12 Hz 13–30 Hz 25–45 Hz	Frontal asymmetry alpha, Beta and Gamma power	LDA	Acc: 85.6%	Quantifying two levels of stress had a better accuracy than three levels (85.6% vs. 58.4%).
[111]	2018	10	4	MIST	Relax–Stress–Neutral	PFC	30 min	Theta, alpha, gamma	Average relative gamma	LDA	Acc: 50.00%	Considering heart rate, skin resistance and trapezius activity features with EEG increased accuracy to 86.00%.
[44]	2018	22	128	MIST	Rest–Three stress levels	PFC	80 min	Delta, theta, alpha, beta, gamma	AP, RP, PL, coherence, relative power ratio, amplitude asymmetry	SVM	Acc: 79.54% Sen: 81.00% Spec: 78.00%	The existence of machine learning techniques provided automatic diagnosis for stress phase.
[56]	2017	25	7	MIST	Control–Stress	PFC	10 min	Delta, theta, alpha, beta	CCA	SVM	Acc: 89.80% Sen: 87.50% Spec: 92.00%	Mental stress was specific and localized to the right ventrolateral PFC.
[45]	2017	22	128	MIST	Control–Stress	Whole scalp	80 min	Delta, theta, alpha1, alpha2, beta1, beta2, beta3, gamma1, gamma2, gamma3	AP, RP, coherence, PL, Amplitude asymmetry	NB SVM	Acc: 94.6% Sen: 98.3% Spec: 93.3% Acc: 93.9% Sen: 96.7% Spec: 92.5%	Using ICA was not recommended with coherence and PL.
[119]	2016	22	7	MIST	Control–Stress	PFC	25 min	Delta, theta, alpha, beta	Mean powers	SVM	Acc: 91.7% Sen: 90.4% Spec: 93.4%	- PFC region was sensitive to mental stress. - Adding fNIRS to EEG improved the accuracy to 95.1%.
[20]	2015	12	8	MIST	Rest–Three stress levels	PFC	60 min	Delta, Theta, Alpha, Beta, Noisy gamma, Noisy signal (64–128Hz)	PSD, energy, average power	SVM	Acc: 94.00%	Increasing difficulty level reduced subject engagement with the stress task.
[79]	2015	5	7	MIST	Control–Stress	PFC	17 min	Alpha	Alpha PSD	SVM	Acc: 95.00%	Alpha rhythm and oxygenated haemoglobin had negative correlation under stress.
[130]	2021	25	7	Mental arithmetic	Control–Stress	PFC	15 min	Alpha	PSD, coherence	-	-	EEG alpha rhythmic demonstrated significant decrease in brain functional connectivity.
[88]	2020	66	19	Mental arithmetic	Relax–Stress	Temporal, Frontal, Central, Occipital, Parietal	4 min	Beta, Alpha, Theta, Delta	MDF, MFMD, SM, RMS, AR	linear SVM Cubic SVM KNN LDA	Acc: 99.70% Acc: 99.75% Acc: 99.93% Acc: 99.94%	Highest accuracy achieved using only two frontal brain electrodes.
[62]	2018	1	14	Mental arithmetic	Rest–Stress	Frontal lobe	31 min	Delta (0.5–3Hz), Theta (3–8Hz), Alpha (8–12Hz), Beta (12–25Hz)	Self-Entropy, MI, Conditional MI	RF	Acc: 97.50%	Combining EEG connectivity with cardiorespiratory features offered high accuracy.
[112]	2016	6	14	Mental arithmetic	Neutral–Three stress levels	Prefrontal, Frontal lobes	20 min	Delta, theta, alpha, beta, gamma	IMF, instantaneous frequency (using HHT)	SVM KNN LDA	Acc: 89.07% Acc: 72.67% Acc: 70.17%	The highest classification accuracy was detected in alpha band.
[23]	2011	5	19	Mental arithmetic	Relax–Stress	Whole scalp	11 min	Delta, theta, alpha, beta	Welch, Yule walker, Burg methods	ANN	91.17%	Maximum classification accuracy achieved when using burg extraction method.
[123]	2019	14	3	Mental arithmetic, SCWT	Relax–Stress	Left, right hemisphere	34 min	Low (0.04–0.15Hz) High (0.15–0.4Hz)	Normalized band power, Power asymmetry	SVM	Acc: 77.90% Spec: 72.00% Sen: 84.60%	Combining HRV with EEG increased accuracy up to 87.50%.
[19]	2019	17	14	Mental arithmetic, videos, playing games	Relax–Two stress levels	Frontal, Occipital lobes	31 min	Delta, theta, alpha, beta	Shannon entropy, mutual information, covariance, precision	RF LR	Acc: 84.30% Acc: 84.30%	Adding HRM and EDA to EEG measurements achieved better accuracy.
[102]	2016	10	14	Mental arithmetic, SCWT	Rest–Two stress levels	Frontal, Occipital lobes	18 min	Theta, alpha, beta	PSD, relative difference of alpha and beta power	SVM	Acc: 96.00%	Providing temporal sliding window with different overlapping increased the accuracy.
[131]	2020	227	62	Psychological labelling	Relax–Stress	PFC	16 min	2.5–3 Hz 24–24.5 Hz 24.5–25 Hz 26–26.5 Hz	PSD	RF	Acc: 81.33% Spec: 80.33% Sen: 82.33%	People who are more stressed, had higher spectral capacity in the left prefrontal cortex.
[108]	2020	33	5	Psychological labelling	Rest–Stress	Frontal, Temporal lobes	39-58 min	Delta, theta, alpha, beta, gamma, slow (4–13Hz), low beta (13–17Hz)	Neural oscillatory features, alpha and beta asymmetry.	SVM NB KNN LR MLP	Acc: 85.20% Acc: 80.79% Acc: 65.96% Acc: 85.15% Acc: 85.13%	- SVM was best in detecting long-term stress when used with alpha asymmetry feature.
[54]	2018	28	1	Psychological labelling	Rest–Stress	Frontal lobe	3 min	Low beta, high beta, low gamma	Neural oscillatory features	SVM	Acc: 78.57%	Using correlation-based feature subset selection method with SVM gave higher accuracy.
[109]	2017	28	1	Psychological labelling	Rest–Stress	Frontal lobe	3 min	Low beta (13–17Hz) High beta (18–30Hz)	Low beta waves, linear regression	NB	Acc: 71.4%	- NB took less computational time comparing to SVM and MLP. - Low beta can be used as feature to quantify stress.
[90]	2019	32	32	Music videos	Relax–Stress	Frontal lobe	-	Delta, theta, alpha, beta, gamma	Nine statistical features, Hjorth parameters, energy, standard deviation, wavelet sum of entropy, PSD.	KNN	Acc: 73.38%	- Using appropriate processing techniques enhances performance. - Final performance can be affected by comparative analysis of scalp sources.
[92]	2018	32	32	Music videos	Relax–Stress	Frontal lobe	40 min	Delta, theta, alpha, beta, gamma	Six statistical features, PSD, HOC, Hjorth parameters, Frontal Asymmetry Alpha.	KNN	Acc: 67.08%	GA-based method outperformed PCA in stress detection and gave better classification accuracy using KNN classifier.
[69]	2018	26	9	Audio-visual stimuli	Relax–Stress	PFC	10 min	Theta, alpha, beta(1,2,3,4,5)	PSI, DTF, GPDC	KNN + SVM	Acc: 90.62%	The effect of stress on brain regions connectivity depends on the subject.
[51]	2011	26	8	Exam stress	Relax–Stress	Whole scalp	6.30 min	2–32 Hz	Higuchi’s fractal dimension, Gaussian mixtures of EEG spectrogram, MSCE.	KNN SVM	Acc: 90.00% Acc: 90.00%	MSCE provided a promising inter-subject validation accuracy (90%) in classifying the EEG.
[132]	2018	11	14	Working hazards and tiredness	Low stress–High stress	Frontal, Occipital lobes	Several hours	Delta, theta, alpha, low beta, beta, high beta, gamma	Seven frequency features, 17 time domain features	KNN GDA SVM	Acc: 65.80% Acc: 74.92% Acc: 75.90%	- KNN showed low accuracy because of inductive bias of KNN method.
[118]	2018	23	30	TSST	Relax–Stress	Frontal, Parietal, Temporal lobes	13 min	Delta, theta, alpha, beta	Transitivity, modularity, path length, efficiency	SVM (beta wave)	Eyes-opened Acc: 92.31% Eyes-closed Acc: 93.62%	- Salivary cortisol level increased during TSST. - Classifier accuracy arised from alpha and beta bandwidths of EEG.
[11]	2015	9	14	SCWT	Low stress–High stress	Frontal, Occipital lobes	12 min	Theta, alpha, beta	PSD, FD, Six statistical features.	SVM KNN	Acc: 85.17% Acc: 76.72%	The followed methodology provided real-time EEG-base stress recognition.
[125]	2019	24	2	SCWT	Relax–Stress	Frontal lobe	11 min	Theta, delta, alpha, beta	PSD	CNN	Acc: 87.50% Sen: 87.50% Spec: 84.00%	Adding HRM, RESP and EDA to EEG will increase stress detection accuracy.
[133]	2020	20	14	Cognitive task	Low stress–High stress	Frontal, Occipital lobes	30 min	Alpha	Discrete wavelet transform	CNN	Acc: 93.00% Sen: 0.923% Spec: 0.934%	-
[105]	2017	15	1	MAST	Relax–Stress	Forehead position	65 min	Alpha1 (8–9Hz), alpha2 (10–12Hz), Beta1 (13–17Hz), Beta2 (18–30Hz)	Attention, Meditation, PSD	SVM	Acc: 86.00% Sen: 84.00% Spec: 90.00%	Changes in the physiological features were correlated with the trend of salivary alpha amylase.
[124]	2017	9	1	Mental workload + public speaking	Three stress levels	Forehead position	-	Delta, theta, alpha, beta	Energy, mean amplitude, RMS, maximum amplitude, weighted mean frequency	SVM NB	Acc: 77.08 Acc: 83.33%	Adding ECG and EDA features to EEG features would improve accuracy.
[50]	2019	26	9	Long-term psychological task	Relax–Stress	Whole scalp	8 min	Theta, alpha1, alph2, beta1, beta2, beta3, gamma1	PSD, LI, CC, CCA, PSI, GC, DTF, MI, MSCE	SVM KNN NB	94.00% 92.00% 90.00%	Using MI and DTF provided the highest accuracy for stress detection.
[122]	2020	86	16	Driving task	Relax–Stress	Frontal, Occipital lobes	45 min	Theta, alpha, low beta, high beta, gamma	Six time-domain features, three frequency-domain features (PSD)	SVM NN RF	Acc: 90.55% Acc: 87.70% Acc: 85.00%	- Adding feature selection technique (MI, PCA, RSFS) improved classifier accuracy. - Combining SVM with MI achieved best accuracy.

**Note:** SCWT: Stroop Color-Word Task; MIST: Montreal Imagining Stress Task; SMO: Sequential Minimum Optimization; PL: Phase Lag; Acc: Accuracy; Sen: Sensitivity; Spec: specificity; fNIRS: Functional Near-Infrared Spectroscopy; PFC: Prefrontal Cortex; RMS: root mean square; TSST: Trier Social Stress Test; BCI: Brain–computer interface; ECOC: error-correcting output code; FD: fractal dimension; GA: genetic algorithm; NN: Neural network; RF: Random forest; RSFS: Random subset feature selection; SVC: Support vector classification; EDA: Electrodermal activity; HRM: Heart rate monitor; IMF: Intrinsic mode function; HHT: Hilbert-Huang Transform; AR: Asymmetry ration; RER: Relative energy ratio; SC: Spectral centroids; SE: Spectral entropy; ESD: Energy spectral density; FCM: Fuzzy C-mean; FKM: Fuzzy K-means; ESD: Energy spectral density; LI: Laterality index; CC: Correlation coefficient; GC: Granger causality; MAST: Maastricht Acute Stress Test; RMS: Root mean square; AR: Auto regression; EDA: Electrodermal activity; HRM: Heart rate monitor; ANN: Artificial neural network; GDA: Gaussian discernment analysis; IAPS: International Affective Picture System; ENN: Elman neural network; CNN: Convolutional Neural Network.

## Data Availability

Not Applicable.
